# Retroaortic Left Renal Vein: A Case Report

**DOI:** 10.7759/cureus.78087

**Published:** 2025-01-27

**Authors:** William A Huff, David D Swan, Jennifer E Geller, Eric Winn, Stacy Chelf

**Affiliations:** 1 Department of Anatomy, DeBusk College of Osteopathic Medicine, Lincoln Memorial University, Knoxville, USA; 2 Department of Surgery, Thomas Jefferson University Hospital, Philadelphia, USA; 3 Department of Anatomy, Lincoln Memorial University, Knoxville, USA

**Keywords:** anatomic variation, medical education, nephrology, retroaortic left renal vein, rlrv, surgery

## Abstract

A retroaortic left renal vein (RLRV) is the term applied to a left renal vein that passes posterior to the abdominal aorta. Typically, the left renal vein traverses horizontally, passing anterior to the abdominal aorta and inferior to the superior mesenteric artery (SMA), before draining into the medial side of the inferior vena cava (IVC). Here, we present a case highlighting type I of the four distinct retroaortic variations observed in the left renal vein. Its prevalence is often underrepresented in medical education despite a clinically significant prevalence in the general population. It is crucial to possess preoperative awareness of this variant to mitigate potential complications linked to vascular damage to the RLRV. These complications encompass severe hemorrhage, renal injury, the necessity for nephrectomy, and, in extreme cases, fatal outcomes during retroperitoneal surgery or interventional procedures. This case report aims to emphasize the significance of incorporating RLRV anomalies into medical education to improve clinical awareness and diagnostic accuracy. The RLRV discussed in this case report was discovered posthumously in an anatomical donor.

## Introduction

During embryonic development, starting around the sixth to eighth week of gestation, the renal veins develop from a network of subcardinal and supracardinal venous systems. Initially, this network consists of multiple channels for venous drainage from the developing kidneys. The left renal vein, in typical anatomy, is formed by the convergence of multiple smaller veins that drain the left kidney, including the left gonadal vein, the left adrenal vein, and various lumbar veins. Once formed, the left renal vein passes anterior to the aorta and posterior to the superior mesenteric artery (SMA) before ultimately draining into the inferior vena cava (IVC), allowing blood to return to the heart. The left renal vein is a unique structure and is unlike the right renal vein. The left renal vein typically traverses horizontally toward the medial side of the IVC. Its trajectory, extending from the hilum of the left kidney to the IVC, involves passing in front of the aorta and below the SMA near each respective vessel. Both renal veins eventually drain to the IVC. However, there are instances of anatomical variation in renal vasculature, most notably in the left renal vein. A left renal vein that passes behind the abdominal aorta is known as a retroaortic left renal vein (RLRV), a relatively rare anatomical anomaly with clinical significance [1].

## Case presentation

**Figure 1:**
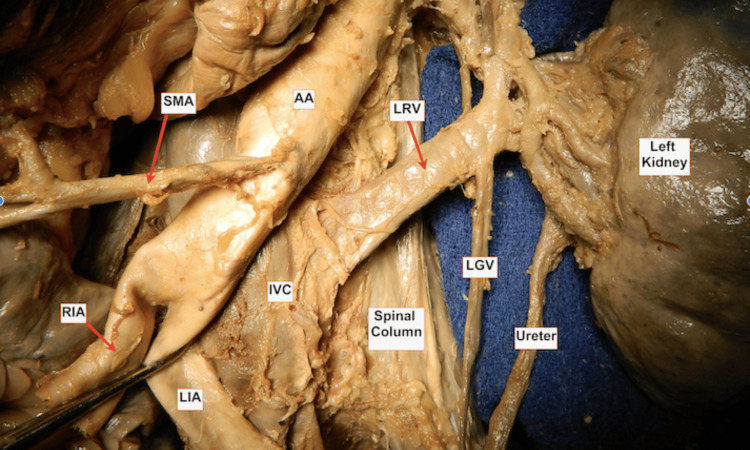
Left kidney and renal vasculature in situ (anterior view) AA: abdominal aorta; SMA: superior mesenteric artery; IVC: inferior mesenteric artery; LRV: left renal vein; LGV: left gonadal vein; RIA: right iliac artery; LIA: left iliac artery

Herein, we describe a case report of a type I RLRV discovered posthumously in a 71-year-old male anatomical donor who gifted his body to the Lincoln Memorial Anatomical Sciences Department for teaching purposes. Routine dissection of the abdominal cavity showed a type I RLRV, where the left renal vein was located posteriorly to the abdominal aorta and anterior to the vertebral column. The size of the left kidney measured 123 mm x 61 mm x 58 mm and was grossly symmetrical with the right (Figure 1). The genital examination revealed a circumcised penis with no abnormalities, including the absence of varicocele or testicular anomalies. The bladder appeared normal in both size and morphology, and the ureters followed their expected course from the ureteropelvic to the ureterovesical junctions. Bilateral renal atrophy was noted, though the renal capsules, parenchyma, and pelvises were grossly unremarkable. An accessory renal artery supplying the inferior pole of the left kidney was identified. Additionally, there were no signs of abdominal or inguinal hernias.

Limited information was provided about the donor’s medical history. The “cause of death” listed Alzheimer’s dementia, chronic obstructive pulmonary disease (COPD), and adult failure to thrive. This donor had a medical history without RLRV-related pathologies such as epigastric pain, varicocele, nutcracker syndrome, pelvic congestion syndrome, hematuria, low-back pain, or renal ectopy [2]. Unfortunately, the donor was returned to the family before additional information and exact morphologic specifications could be gathered. 

## Discussion

There are several variations in renal venous anatomy, and four classes are specific to the left renal vein. This type of renal variation is called an RLRV [3]. A type I RLRV describes an obliterated ventral preaortic limb of the left renal vein with a patent retroaortic limb that remains and connects with the IVC in its anatomical typical position (L1 and L2). A type II RLRV arises from the obliteration of the ventral preaortic limb, and the remaining dorsal limb turns into an RLRV but connects with the gonadal and ascending lumbar vein at the level of L4 and L5 before joining the IVC. A type III RLRV is the circumaortic left renal vein due to the preservation of subsupracardial and intersupracardial anastomoses and the dorsal limb on the left renal vein. Finally, a type IV RLRV is when the ventral preaortic limb of the left renal vein is obliterated, while the dorsal limb travels inferiorly and obliquely to join the left common iliac vein [4]. RLRV types I, II, III, and IV are found in about 0.3%-1.9%, 0.4%-0.9%, 1.5%-8.7%, and 0.16%, respectively [5]. Previous studies have employed various diagnostic techniques to identify anomalies in the IVC, including autopsy studies, renal venography, color Doppler ultrasonography, computed tomography (CT), and magnetic resonance imaging. However, due to CT technology advancements, multidetector computed tomography (MDCT) has now largely supplanted traditional angiography and venography across numerous clinical scenarios. MDCT stands out as a dependable, user-friendly, and noninvasive method for visualizing abdominal organs and vascular structures [6]. 

While seemingly innocuous, the RLRV can have profound implications if undiagnosed. Although usually asymptomatic, RLRV can present with hematuria, flank pain, scrotal varicocele, and pelvic congestion secondary to nutcracker syndrome [7]. Nutcracker syndrome occurs when the RLRV is compressed, leading to venous congestion in the left kidney. Nutcracker syndrome refers to a condition where the left renal vein is compressed between the aorta and the SMA in the abdomen, causing blood to flow backward and potentially leading to symptoms like flank pain and hematuria. This congestion can lead to renal infarcts, vascular compromise, bacterial colonization, and abscess formation [7]. Other factors that can cause nutcracker syndrome include anatomic variation, low body mass index (BMI), rapid weight loss, pancreatic tumors, or aortic aneurysms [7,8]. 

Recognizing and understanding the existence of the RLRV is crucial for preventing inadvertent complications during surgeries and diagnostic interventions involving renal vasculature. Failure to recognize these anomalies can lead to complications such as severe bleeding, nephrectomy, or even death [8]. Awareness of renal vein anomalies is also critical for differentiating retroperitoneal tumors, lymph node pathologies, and aortic dissections. Careful preoperative review of imaging studies for RLRV can help prevent potentially fatal complications during surgery [9]. In shedding light on the RLRV, this case report aims to contribute to the growing body of knowledge surrounding anatomical variations, advocating for their inclusion in medical education to enhance the preparedness of healthcare professionals and ultimately improve patient outcomes. Incorporating such variations into medical education curricula will create well-versed healthcare professionals to increase the diversity they may encounter in clinical practice. 

This study was limited by the absence of detailed morphological specifications of the RLRV and the lack of comparative data for the right renal vasculature, as the donor was returned to the family before precise measurements could be obtained. Additionally, the single-donor nature of the study restricts the generalizability of the findings, as anatomical variations may differ across larger or more diverse populations. The reliance on postmortem observations also precluded functional assessments or correlation with clinical symptoms. Furthermore, the inability to perform advanced imaging, such as CT or venography, limits the detailed characterization of the vascular anatomy. These limitations underscore the need for further studies with larger sample sizes and comprehensive imaging to enhance our understanding of RLRV and its clinical implications.

This case report was conducted in accordance with institutional policies and ethical guidelines. The anatomical donors used in this study had provided prior documented consent for the use of their bodies in research and education through the Lincoln Memorial University-DeBusk College of Osteopathic Medicine Anatomical Donation Program. As no identifiable information was included and the research involved deceased individuals, this study met the criteria for exemption from the Institutional Review Board (IRB) review under 45 CFR 46.104(d).

## Conclusions

RLRV is usually asymptomatic but can occasionally present with symptoms such as hematuria, flank pain, or vascular dilations like varicocele. Type I is the most common anatomical variant of RLRV. Recognizing undiagnosed RLRV is crucial, as it can have significant clinical implications and may alter the surgical approach in intraabdominal and urological procedures. This case report underscores the importance of identifying anatomical variations like RLRV and advocates for their inclusion in medical education to enhance the preparedness of healthcare professionals and ultimately improve patient outcomes.
